# Social and Built Environments Related to Cognitive Function of Older Adults: A Multi-Level Analysis Study in Taiwan

**DOI:** 10.3390/ijerph18062820

**Published:** 2021-03-10

**Authors:** Hui-Chuan Hsu, Chyi-Huey Bai

**Affiliations:** 1 School of Public Health, College of Public Health, Taipei Medical University, 11031 Taipei, Taiwan; baich@tmu.edu.tw; 2 Research Center of Health Equity, College of Public Health, Taipei Medical University, 11031 Taipei, Taiwan; 3 Department of Public Health, College of Medicine, Taipei Medical University, Taipei 11031, Taiwan

**Keywords:** age friendly city, built environment, cognitive function, social environment, older adults

## Abstract

The purpose of this study was to examine the associations between cognitive function, the city’s social environment, and individual characteristics of older adults. The individual data of older people were from the Nutrition and Health Survey in Taiwan 2013–2016. The participants who were aged 65 and above were included in the analysis (*n* = 1356). City-level data were obtained for twenty cities in Taiwan. The data of city-level indicators were from governmental open data and Taiwan’s Age Friendly Environment Monitor Study. A multilevel mixed-effect model was applied in the analysis. Population density, median income, safety in the community, barrier-free sidewalks, high education rate of the population, low-income population rate, household income inequality, and elderly abuse rate were related to cognitive function in the bivariate analysis. When controlling for individual factors, the city’s low-income population rate was still significantly related to lower cognitive function. In addition, the participants who were at younger age, had a higher education level, had a better financial satisfaction, had worse self-rated health, had higher numbers of disease, and had better physical function had better cognitive function. Social and built environments associated with cognitive function highlight the importance of income security and the age friendliness of the city for older adults. Income security for older people and age-friendly city policies are suggested.

## 1. Introduction

The prevalence of dementia increases with age; the prevalence of dementia in 2017 increased by age from 2% to 41% for people aged 65 to 90 and above [1]. In Taiwan, approximately 18.16% of older adults have mild cognitive impairment, and 7.78% of older people have dementia [2]. Cognitive function impairment and dementia have great impacts on individuals, their families, and society. Due to its progressive nature, dementia is usually irreversible, thus preventing cognitive decline should be involve greater effort than a simple treatment-oriented approach. The characteristics and risk factors of individuals have been examined in previous research, including age, sex, genes, education, socioeconomic status, chronic disease, physical disability, depressive symptoms, stress, exercise, smoking, nutrition and dietary behaviors, living arrangement, social network, and social participation [3,4,5,6,7,8,9,10,11,12,13,14,15,16,17,18,19,20]. In addition, environmental factors have been found to be relevant to cognitive function, such as air pollution and temperature. However, the effects of the social environment are less well-explored. In this study, we focus on the associations between the social environment and cognitive function of older adults.

### 1.1. Theoretcial Explanation

From the ecosystem perspective [21], the microsystem (individual factors), mesosystem (interpersonal relationship), exosystem (living environment), and macrosystem (policy and social context) may influence an individual’s health and wellbeing. Environmental effects include compositional effects and contextual effects [22]. The compositional effects indicate the heterogeneity of areas, and such area differences affect the health outcomes of individual differences. Contextual effects refer to the social and physical environment with which individuals interact and accordingly affect health. In public health, physical and social environments can be characterized as physical characteristics, accessibility to household/work setting/leisure environments, health/social services support, the social and cultural quality of the neighborhood (such as social capital), and the psychological effect of the area reputation or infrastructure differences [22,23]. The social environment is not just a geographical area but also an outcome of socioeconomic status, public services, human behaviors, and culture. Such social characteristics last longer and may have a stable influence on the residents in the area [24]. The social environment should be viewed as comprising social epidemiological factors that are risky or protective for an individual’s health.

### 1.2. Cognitive Function and Individual Factors

Some demographic variables are related to better cognitive function among older adults, including being younger, male, married, higher educated, higher household income or better socioeconomic status [3,4,8,9,12,17], living in an urban area, and more social participation [12]. There are also ethnicity differences in cognitive function [3,4,8,17].

Some genes have been found to be related to cognitive function, such as Apoliprotein E (APoE) [6,7] and several serum microRNAs [11]. Certain diseases are often comorbid with cognitive impairment, such as hypertension, diabetes, heart disease, stroke, etc. [8,9]. Having more physical disabilities is often related to poorer cognitive function [5,8]. Cognitive function is also related to more depressive symptoms among older adults [8,9].

Lifestyle is related to cognitive function. Regular exercise is found to be beneficial for better cognitive function [4,9,14,20]. The association of dietary patterns and cognitive function is not consistent. Consuming more vegetables [6] and a Mediterranean diet [15] is related to better cognitive performance and delaying the decline of cognitive function for older adults. In other research, dietary patterns are not significantly related to cognitive function [16]. Consistent drinking was related to poorer cognitive function in a longitudinal study [20], and another study indicated that a higher intake of red wine was related to a higher incidence of Alzheimer’s disease [7].

### 1.3. Cognitive Function and Environmental Factors

Recent research has focused on the effect of the environment on cognitive function. A systematic review [25] categorized environmental influences as follows: socioeconomic status of the community, demographic composition of the community, social abnormality (such as crime rate), and social climate or social connectedness.

Nature and the physical environment, such as particulate matter 2.5 (PM_2.5_) [3,18] and temperatures that are too high or too low [19], may affect cognitive function. Empirical studies have found a relationship between social environment and cognitive function. The pressure of the community (such as the reputation of the community, decayed industry, and junk) in connection with air pollution may have a negative effect on cognitive function [3]. Social fusion and abnormalities (such as the safety of walking at night, abandoned spaces, cleanness, and graffiti) in the neighborhood may also be related to the cognitive function of older adults [8].

Associations between the built environment and cognitive function have been explored. Lower neighborhood socioeconomic status, older, nonwhite races, lower population density, more decayed public spaces, inconvenient living function, and lower mixed-land use have been found to be related to lower cognitive function [25,26]. The better walkability of sidewalks [9], more barrier-free public space, more bus lines, and more employment service in the community [12] are associated with better cognitive function in older adults. It is possible that better opportunities for social participation and social support in the community are beneficial for the cognitive function of older adults. A longitudinal Irish study [5] showed that older adults living in towns with a moderate population density have better cognitive function. Other English studies indicated that higher accessibility to the natural environment may be associated with lower education, although a natural environment is good for reducing depression and anxiety [26,27]. Therefore, appropriate resources and stimulation may be beneficial for cognitive function. However, in older adults who are disabled or living with dementia, a complicated environment might be a burden on cognitive function and emotional health.

The composition of the social environment may also be related to cognitive function. Age composition may represent the industry types and health resources [8]. A higher lower-income rate among the population of the community suggests a poorer cognitive function [9]. A previous study in Taiwan reported that communities with a lower education level, a greater aging population, and more unemployment and single-parent families influence poorer health conditions [28], but associations between cognitive function and environmental factors were not examined. The socioeconomic status and cognitive function (especially semantic memory) of the communities may be related to longitudinal cognitive decline, but such a relationship is no longer significant when controlling for other variables [13]. The possible mechanism is that the socioeconomic status of the community may influence interactions and stimulation of cognitive function and thus delay the decline in cognitive function in the long term. However, another study did not find supportive evidence for the effect of community deprivation on cognitive function [26].

Although some aspects of the relationship between environmental factors and cognitive function have been explored, the results are mixed and many environmental factors may influence cognitive function. In particular, the mechanism of the effects of the social environment on the cognitive function of older adults needs further investigation. The purpose of this study was to examine the associations between social environmental factors, individual factors, and the cognitive function of older adults in the community.

## 2. Materials and Methods

### 2.1. Data and Sample

The individual data were from the Nutrition and Health Survey in Taiwan (NAHSIT) 2013–2016 conducted by the Health Promotion Administration, Ministry of Health and Welfare, Taiwan, R.O.C. NAHSIT is a nationwide, cross-sectional survey on health, dietary, and nutritional status in all ages in the general population. A three-stage probability proportional to size sampling plan was performed in 160 primary sampling units which cover 20 cities and 359 townships or city districts. Under each primary sampling unit, stratified sampling based on age and sex was used to guarantee the distributions. The sample was representative at the nation and city levels. To omit the period affect, participants in different cities were interviewed in turn from 2013 to 2015. Detailed information is described elsewhere [29]. Data collection was conducted based on door-to-door face interviews of well-trained interviewers using standardized questionnaires and protocols. Only those who could be communicated with and responded to the interviewers were included as the sample of NAHSIT. Participants who had Alzheimer’s disease or severe diseases, were pregnant or breast feeding, who had hearing and visual impairments, and who were living in institutions were excluded from the questionnaire interview. In total, 11,072 persons completed the survey. In this study, only participants aged 65 years and above were included for analysis. The complete sample for this study was 1356 persons.

The data of the city-level indicators were from the open data of the government, and the data years were confined to 2013 to 2015. If the data year was not available for the indicators, the closest available data were then applied. The age-friendly city indicators were from Taiwan’s Age Friendly Environment Monitor Study [30], which collected city-based area-level indicators from 2016 to 2018. The 2016 data of the selected indicators were applied.

### 2.2. Ethical Consideration

The data used in this study were subjected to a deidentification process when they were released. Approval of the Taipei Medical University Joint Institutional Review Board (N201912135) was obtained before the study was conducted.

### 2.3. Measures

#### 2.3.1. Dependent Variable

The dependent variable was measured using the Mini-Mental State Examination (MMSE) [31]. The total score ranged from 0 to 30. The typical cut-off point for cognitive impairment is a score lower than 25, or the cut-off point is defined by education. In this study, we used the continuous MMSE score to measure cognitive function.

#### 2.3.2. Independent Variables: Individual-Level

The individual-level-related factors included demographics, socioeconomic status, health behavior, and health conditions, data which were all from the questionnaires. Demographic and socioeconomic status variables included age, sex, education (illiterate, informal, elementary school, primary high school, senior high school, college/university or above), marital status (having a spouse or not), working status (yes/no), monthly income (ordinal), and subjective financial satisfaction (score of 1 to 4, indicating very low satisfaction to abundance of wealth).

Health behaviors included smoking (nonsmoker, ex-smoker, and current smoker), drinking alcohol (nondrinker, social drinker, and frequent drinker), dietary pattern, and physical activity level. The dietary pattern was defined based on the results of a food intake questionnaire. Food intake was assessed by the Food Frequency Questionnaire (FFQ) [32], which is a common tool to measure the dietary intake. The frequency of food intake over a week was assessed. We used 56 food items from the data to identify dietary patterns (please see the analysis section). Because one of the clusters contained too few participants (*n* = 3), this dietary pattern cluster was combined with a similar group. Finally, three dietary patterns were identified: low protein and high vegetables (72.6%), high protein and high calories (18.6%), and low vegetables/fruits and high cake/cookies (8.8%). Physical activity level was measured by the frequency and hours of activities per week and the metabolic equivalents were calculated. Then, the physical activity level was defined as one of three levels according to International Physical Activity Questionnaire (IPAQ) [33]: low, moderate, or high. Because few people in this sample were in the high group, the physical activity level was recoded into two groups: low and moderate.

Health condition variables included self-rated health, physical function, disease number, negative emotions, and positive emotions. Self-rated health was measured based on three items: how do you rate your health, how is your health now compared with it 1 year ago, and how is your health compared with others at your age. Each item was scored from 1 to 5, and the total score ranged from 3 to 15. Disease number was the cumulative number of morbidity of the following diseases: cataract, glaucoma, tuberculosis, emphysema, chronic bronchitis, digestive ulcer, irritable bowel syndrome, fatty liver, liver cirrhosis, abnormal thyroid, gout, arthritis, hypertension, hyperlipidemia, diabetes, kidney disease, calculus, heart disease, Parkinson’s disease, dementia, depression, anxiety, psychiatric disease, urinary incontinence, prostate enlargement, cancer, and others. Physical function was measured according to Nagi’s disability scale [34], which measures advanced physical functioning activities. Each item was scored 1 to 3, and the total score ranged from 3 to 30; a higher score indicated a better physical function.

Emotion was measured on the basis of the occurrence of nine items in the past month—namely, feeling nervous, frustrated, depressed, exhausted, tired, active, energetic, calm, and happy. Each item was scored from 1 to 6, indicating a frequency from never to always.

#### 2.3.3. Independent Variables: City-Level

Area-level variables were mainly obtained from the open data or the existing secondary data during 2013–2016. The area-level indicators were city-based variables.

1. City indicators from the governmental open data

The open data year was defined from 2013 to 2015, and the average of the three years was used. These indicators included population density (defined as the average persons per square kilometer), elderly percentage (defined as the percentage of older people among the entire city population), particulate matter 10 (PM_10_) density, medical professionals (the number of medical professionals per 10 thousand people), hospital beds (the number of hospital beds per 10 thousand people), crime rate (the average crime rate), low income percentage, median disposable income (NT dollars) of the household, unemployment rate, public library density (the number of public libraries per 100 thousand people), high education level (the population with a college/university education level or above in the city), household income Gini coefficient (the household income of the city residents was sorted into 5 groups and then the Gini coefficient was calculated; a higher Gini coefficient indicated a more diverse income range among the lowest and the highest), and green area for leisure purposes (number of hectares per 10,000 persons).

2. Age-friendly city indicators

Most of the age-friendly city indicators were self-reported; some were from government statistics. The age-friendly city indicators in 2016 included barrier-free sidewalk percentage, barrier-free public building entrance percentage, barrier-free pathway outside of residence, satisfaction with the distance from home to the bus stop, barrier-free parking percentage, barrier-free bus percentage, step-entrance bus percentage of all the buses, bus accessibility percentage, housing affordability (residence cost less than 30% of disposable income), feeling safe in the community, elderly abuse rate (person-time/100 persons), nonpoverty rate percentage, social activity participation rate, lifelong learning rate, leisure and exercise participation rate, voting rate, volunteer participation rate, Internet use rate, community center density (community center number per 10,000 persons), and utilization rate of free health check-ups for older people.

### 2.4. Analysis

The analysis was conducted using IBM SPSS software version 22.0 (IBM, SPSS Inc., Chicago, IL, USA). Descriptive analysis, Pearson’s correlation, t test, one-way ANOVA, factor analysis and cluster analysis were conducted for variable processing. Dietary patterns were measured with a food frequency questionnaire in which 56 food items were first categorized into 16 types of foods using factor analysis (please see Supplementary Table S1), by principal component analysis and Varimax rotation. Then, a two-stage cluster analysis (hierarchical cluster and K-means cluster) was conducted to determine the best cluster numbers of dietary patterns. The 16 food-intake types of the participants were classified into 4 dietary patterns via cluster analysis (Supplement Table S2). Because one of the clusters contained too few participants (*n* = 3), this dietary pattern cluster was combined with a similar group. Finally, three dietary patterns were identified: low protein and high vegetables (72.6%), high protein and high calories (18.6%), and low vegetables/fruits and high cake/cookies (8.8%). The 9 emotion items were also classified into two factors by factor analysis: positive emotion (active, energetic, and happy) and negative emotion (the remaining six items). The scores were summed to define a positive emotion and a negative emotion score.

The correlations of individual-level and city-level variables with cognitive function score were examined using Pearson’s correlation. Then, a multi-level analysis by the mixed linear model was conducted. Fixed effects of individual factors and city factors and random effects from intercept and within city were assumed.

## 3. Results

Table 1 shows a description of the sample characteristics. The average age was 73.43 years old and the sex percentage was nearly equal. The average cognitive function based on the MMSE score was 23.18 and the percentage of scores lower than 25 (mild cognitive function) was 39.9%. The dietary patterns of the older participants are shown in the Supplementary Data. Table S1 shows a factor analysis of the food frequency intake in a week for older adults. Through factor analysis, 16 types of food were extracted. Next, a cluster analysis of the 16 types of food was conducted to identify dietary patterns. The resulting dietary patterns are shown in Supplementary Table S2. Four dietary patterns were identified in the beginning. Due to the small number of cases in the ‘high protein and low vegetables’ group (*n* = 3), this group was combined with the similar group ‘high protein and high calories’. Thus, in total, three types of dietary patterns were identified: low protein and high vegetables (72.6%), high protein and high calories (18.6%), and low vegetables/fruits and high cookies/sweet drinks (8.8%). Table 2 shows a description of the city-level indicators. Twenty cities/counties in Taiwan were evaluated.

Table 3 shows correlations between the cognitive function score and the city-level indicators. Only the significant indicators are listed in the table. Cognitive function was significantly positively related to higher population density, median income, safety in the community, barrier-free sidewalks, a high education rate of the population, a lower low-income population rate, lower household income Gini coefficient (i.e., more equal income distribution), and lower elderly abuse rate. Among the nine city-level indicators, some were highly correlated: population density, income Gini coefficient, high education, and median income. To prevent collinearity of these city-level indicators, we only chose seven indicators in the following analysis. 

Table 4 shows the results of linear mixed models of the associations of city- and individual-level factors with the cognitive function of older adults. Model 0 was the null model, with only intercept and random effects included. Model 1 included only the individual-level factors and random effects. Older adults who were younger than 80 years old, were well-educated, were more satisfied with their financial status, had worse self-rated health, had a higher number of diseases, and had better physical function showed higher cognitive function. Model 2 included only the city-level indicators and the random effects. Cities with a smaller low-income population (β = −0.641, *p* < 0.01) were associated with better cognitive function. Model 3 included both individual-level and city-level variables. Living in a city where the low-income population was high was associated with worse cognitive function (β = −0.544, *p* < 0.01). In addition, older adults who were younger, were well-educated, had better financial satisfaction, had worse self-rated health, had a higher number of diseases, and had better physical function had better cognitive function.

## 4. Discussion

This study examined the associations between the cognitive function of older adults and individual- and city-level factors in Taiwan. In the bivariate analysis, better cognitive function was related to higher city population density, higher median income, higher safety in the community, more barrier-free sidewalks, a more well-educated population, a smaller low-income population, more income inequality, and a lower elderly abuse rate. When controlling for individual factors in the mixed-effect model, cities with a smaller low-income population was still significant. In addition, the age, education, financial satisfaction, and health status of older adults were also significantly related to the cognitive function of older adults.

### 4.1. City Factors and Cognitive Function

The low-income population rate in the neighborhood was negatively associated with cognitive function, which is consistent with previous research [9,25]. Thus, when a city has a larger low-income population, the living standard in the city is lower or the population in the primary industry is greater. In lower-income cities, social interaction may be less frequent and city development may be slower. Therefore, the stimulation of cognitive function (especially for older adults) is lower and cognitive function is less developed or declines faster. A low-income population was also related to higher income inequality (higher income Gini coefficient) in this study. However, higher income inequality was related to better cognitive function, although not significantly in the final model. In fact, the low-income rate was also highly related to population density and a well-educated population. A possible explanation is that income is more unequal in bigger cities, and bigger cities promote more interactions, which is good for cognitive function. In addition, previous research found that a too high or too low population might be associated with lower cognitive function [5]. In fact, we also have tried to define population density as a categorical variable (low/medium/high); please see Supplementary Table S3. The medium population density group had higher cognitive function than the lower and higher population density cities, but the relationship was not significant.

Better cognitive function was associated with more safety in the community, more barrier-free sidewalks, and a lower elderly abuse rate in the bi-variate analysis, although the associations were not significant in the multi-level analysis. A safer community and more barrier-free sidewalks for leisure reduce the pressure of daily life for older adults and may encourage older adults to engage in social participation and physical activity outdoors [9,35]; these factors are all beneficial for cognitive function.

Previous research suggests that age composition and unemployment of the environment may be related to cognitive function [8,28]. In this study, the cities with a higher percentage of older people or a higher unemployment rate were not significantly related to individual’s cognitive function. It is possible that the effects of these factors were cumulative for cognitive function, but the data were cross-sectional and only measured at one time. Past research also suggests the built environment of age-friendly cities may be related to cognitive function [9,12,25,26], but most of the built environment factors were not significant in this study. It is possible that we can only measure by city as an area unit, and there is built environment variability within a city; thus, the effects of built environment were diluted.

### 4.2. Individual Factors and Cognitive Function

Regarding individual characteristics, having a worse physical health status (self-rated health and more diseases) was related to better cognitive function, while having better physical function was also related to better cognitive function. Self-rated health is a general rating of personal health, but not specifically related to cognitive function. The chronic disease number may gather the impacts of all diseases together, but not all diseases are related to cognitive impairment. In addition, some certain neurodegenerative disorders that may be related to mild cognitive impairment, such as Parkinson’s disease [36], were not measured in this study. Physical function disability is usually accompanied by lower cognitive function [35], and thus a lower cognitive function may be a predictor for physical disability and further demands of long-term care.

Social support and social network may provide social interactions to stimulate cognitive function and are also good for physical and psychological health [22,23]. We only analyzed the effect of marital status, and having a spouse was associated with higher cognitive function at the borderline significant level. The variables about social support and social interaction were unavailable in this data.

Dietary patterns were expected to be related to cognitive function but not significant in this study. One possible explanation is that a long-term dietary pattern may affect cognitive function, but this study only examined the associations in the cross-sectional data. The other explanation is that only specific food items were closely to cognitive function, and the dietary pattern mixed up the effects of all the food items with cognitive function. Thus, the dietary pattern was not significant.

### 4.3. Limitations

There are some limitations in this study. First, the data were from secondary databases. Some of the variables were not available in this study. For example, the open data of the government only provides PM_10_ but not PM_2.5_ statistics in the data year. Some of the city-level variables obtained from other surveys (such as social trust and happiness in the cities) did not cover all the cities in Taiwan and thus could not be combined. Second, the data for individuals were cross-sectional. Repeated measures of changes in the cognitive function of older adults were not observed. The causal relationship between city indicators and cognitive function was not confirmed. However, we tried to use the closest year of city indicators to make the associations reasonable. Third, city was used as the area-level unit because only city-level indicators were available. There may be community variations in a city. Due to the human data privacy law in Taiwan, only city-level variables can be released but not further smaller units in the open data or the released secondary data. The city area may be larger than the life circle of older adults. However, city is the unit used for old-age policies of local governments. Thus, using city as the unit to define area-level indicators is suitable for this topic. Fourth, the data were obtained from certain years in Taiwan. The results may not be generalized to other places, nor can the results reflect the most recent changes, such as air pollution and the effects of the latest active aging policies.

## 5. Conclusions

City-level indicators may be related to cognitive function and are especially related to financial security and city development. The development of a city is not only related to economics but also to people’s living standards and opportunities for social interaction, which eventually affect the health of people of all ages. We suggest that the income security policy should be strengthened. In Taiwan, the pension system is under reform, and income security may be reduced for pensioners, not to mention some older people who are under no or little pension protection. Regarding the issue of neighborhood poverty, local governments should empower the community and encourage partnerships with nonprofit organizations and social enterprises to promote community development and to rebuild a safe and supportive environment in communities at risk of poverty. In addition, a decent, safe, age-friendly city with proper social interactions and social support may be beneficial for the cognitive function of older adults. Age-friendly city policies are generally implemented in local governments of the cities or counties in Taiwan. Planning for convenient and barrier-free public transportation and sidewalks, safe and barrier-free environments in the community, and supportive community services to encourage social participation and social support for older adults are suggested as age-friendly city policies.

## Figures and Tables

**Table 1:** Descriptive analysis of the sample.

Variable	*N*	Mean (SD) or %
Sex		
Male	677	49.9%
Female	679	50.1%
Age	1356	73.43 (0.49)
Age 65–69	464	34.2%
Age 70–74	355	26.2%
Age 75–79	268	19.8%
Age 80+	269	19.%
Education		
Illiterate	175	12.9%
Informal or elementary school	634	46.9%
Primary high school	170	12.5%
Senior high school	177	13.1%
College or university and above	199	14.7%
Marital status		
No spouse	442	32.6%
Having spouse	913	67.4%
Work		
No	1077	79.4%
Yes	279	20.6%
Monthly Income	1272	3.63 (2.21)
<NT 10,000	479	37.7%
NT 10,000–19,999	485	3.1%
NT 20,000–49,999	208	16.4%
NT 50,000+	100	7.9%
Financial satisfaction		
Very difficult	119	9.0%
Difficult	308	23.3%
Just make	726	55.0%
Abundant	168	12.7%
Smoking		
Non-smoker	919	70.0%
Ex-smoker	280	21.3%
Current smoker	114	8.7%
Drinking alcohol		
Non-drinker	757	55.8%
Social drinker	418	30.8%
Frequent drinker	181	13.3%
Dietary pattern #		
Low protein and high vegetable	964	72.6%
High protein and high calories	247	18.6%
Low vegetable/fruits and high cookies/sweet drinks	117	8.8%
Physical activity IPAQ		
Low	1287	94.9%
Moderate	53	3.9%
High	16	1.2%
Self-rated health (3~15)	1227	8.10 (2.19)
Disease numbers	1356	2.53 (1.93)
Physical function (9~27)	1216	22.06 (4.78)
Negative emotion (6~33)	1196	13.94 (3.92)
Positive emotion (3~18)	1187	12.02 (4.27)
MMSE score (0~30)	1327	23.18 (8.02)
Impaired (score ≤ 24)	530	39.9%
Intact (score ≥ 25)	797	60.1%

Note: *N* = 1356. Missing cases were not included. NT: New Taiwan Dollars. # Dietary pattern analysis is shown in Supplementary Tables.

**Table 2:** Descriptive analysis of the city indicators (*n* = 20).

City indicators	Mean	SD
Population density (persons square kilometre) (2013–2015)	1616.95	2342.40
Elderly percentage (2013–2015)	12.96	2.06
PM_10_ (2013–2015)	48.14	12.79
Medical professionals/10000 persons (2013–2015)	94.19	33.94
Hospital beds/10000 persons (2013–2015)	73.78	25.96
Crime rate (2013–2015)	1195.01	249.11
Low income percentage (2013–2015)	1.68	1.11
Median disposable income (NT dollars) (2013–2015)	762,998.90	160,455.10
Unemployment rate (2013–2015)	3.95	0.09
Public library/100 thousand population density	4.81	9.11
High education population percent (college/university+)	28.3	6.51
Household income Gini coefficients (2013–2015)	0.33	0.03
Barrier-free sidewalk percentage (2016)	0.61	0.22
Barrier-free entrance of public building percentage (2016)	91.65	22.14
Barrier-free pathway outside residence (2016)	23.22	17.04
Bus stop satisfaction (2016)	91.29	8.42
Barrier-free parking percentage (2016)	2.24	1.37
Barrier-free bus percentage (2016)	57.49	30.16
Bus accessibility percentage (2016)	72.14	18.77
Housing affordability percentage (2016)	73.72	9.03
Safety in the community percentage (2016)	95.88	3.02
Elderly abuse person-times/100 persons (2016)	0.20	0.05
Non-poverty rate (2016)	39.52	12.35
Social activity participation rate (2016)	12.98	5.87
Lifelong learning rate (2016)	7.96	3.46
Leisure and exercise participation rate (2016)	6.56	4.08
Voting rate (2016)	89.69	10.90
Volunteer participation rate (2016)	10.86	4.36
Internet use rate (2016)	14.27	8.62
Community care center density/10,000 persons (2016)	0.52	1.43
Use rate of health check-up for older people (2016)	63.58	16.84
Greenland for leisure area per 10,000 persons (hectare) (2017)	6.13	3.83

Note: NT: New Taiwan Dollars.

**Table 3:** Correlation matrix of cognitive function score with area-level indicators.

Variables	Cognitive Function	Population Density	Low Income Rate	Median Income	Income Gini	Safety of Community	Elderly Abuse	Barrier-Free Sidewalk	Higher Education Rate
Cognitive function	1								
Population density	0.079 **	1							
Low-income rate	−0.062 *	−0.140 **	1						
Median income	0.082 **	0.737 **	−0.459 **	1					
Income Gini	−0.061 *	−0.451 **	0.504 **	−0.631 **	1				
Safety of community	0.097 **	0.472 **	−0.373 **	0.413 **	−0.421 **	1			
Elderly abuse	−0.063 *	−0.373 **	0.220 **	−0.377 **	0.284 **	−0.468 **	1		
Barrier-free sidewalk	0.065 *	0.430 **	0.075 **	0.431 **	−0.130 **	0.173 **	−0.477 **	1	
High education rate	0.096 **	0.810 **	−0.332 **	0.944 **	−0.509 **	0.462 **	−0.423 **	0.538 **	1

Note: Analysis by Pearson’s correlation. Only the significant indicators are included. * *p* < 0.05, ** *p* < 0.001.

**Table 4:** City- and individual-level factors associated with cognitive function for older adults in Taiwan in 2013–2016 by a mixed-effect model.

Variable	Model 0	Model 1	Model 2	Model 3
Fixed Effects: Individual-level indicators				
Intercept	25.398 (0.273) ***	15.076 (1.280) ***	12.116 (8.910)	10.478 (6.061)
Age 65–69		1.791 (0.356) ***		1.820 (0.354) ***
Age 70–74		1.634 (0.359) ***		1.680 (0.358) ***
Age 75–79		1.440 (0.372) ***		1.479 (0.371) ***
Sex (Male)		0.372 (0.310)		0.361 (0.310)
Marital status (no spouse)		−0.482 (0.263)		−0.479 (0.262)
Education (ordinal 1–5)		1.317 (0.106) ***		1.298 (0.106) ***
Financial satisfaction		0.328 (0.157)*		0.346 (0.157) *
Smoking (non-smoker)		−0.183 (0.471)		−0.185 (0.469)
Smoking (Ex-smoker)		0.228 (0.467)		0.217 (0.466)
Drinking alcohol (Non-drinker)		0.275 (0.377)		0.160 (0.377)
Drinking alcohol (Social drinker)		0.218 (0.362)		0.127 (0.362)
Dietary pattern (High protein and high calories)		−0.226 (0.297)		−0.240 (0.297)
Dietary pattern (low vegetable/fruits and high cookies/drinks)		0.540 (0.395)		0.513 (0.394)
Physical activity (low)		−0.247 (0.497)		−0.225 (0.497)
Self-rated health		−0.130 (0.063) *		−0.125 (0.063) *
Disease numbers		0.139 (0.062) *		0.144 (0.062) *
Physical function		0.203 (0.031) ***		0.200 (0.031) ***
Negative emotion		−0.003 (0.033)		0.001 (0.033)
Positive emotion		0.053 (0.032)		0.053 (0.032)
Fixed Effects: City-level indicators				
Population density #			0.017 (0.010)	0.002 (0.007)
Low income percent			−0.641 (0.205) **	−0.544 (0.136) **
Average household income Gini			7.330 (9.498)	3.663 (6.366)
Safety in the community			0.109 (0.079)	0.038 (0.053)
Barrier-free sidewalk			1.355 (1.050)	0.947 (0.698)
Elderly abuse rate			1.875 (4.581)	0.353 (3.064)
Random effect				
Residual	19.534 (0.850)	13.111 (0.576)	19.534 (0.850)	13.107 (0.576)
Intercept (city)	1.117 (0.478)	0.360 (0.198)	0.304 (0.259)	0.044 (0.112)
Goodness of fit	-−2RLL = 6273.540	−2RLL = 5850.326	−2RLL = 6252.405	−2RLL = 5835.054
	AIC = 6277.540	AIC = 5854.326	AIC = 6256.405	AIC = 5850.965
	BIC = 6287.499	BIC = 5864.249	BIC = 6266.352	BIC = 5848.965

Note: −2RLL: −2 restricted log likelihood. AIC: Akaike’s Information Criterion, BIC: Schwarz’s Bayesian Criterion. The reference groups: age (age 80+), sex (female), marital status (having spouse), smoking (current smoker), drinking alcohol (frequent drinker), dietary pattern (low protein and high vegetables), physical activity (medium). Other variables were continuous or ordinal. # Population density=persons per square kilometres/100. * *p* < 0.05, ** *p* < 0.001, *** *p* < 0.001.

## Data Availability

We do not have the right to share the NAHSIT data.

## Supplementary Materials

The following are available online at https://www.mdpi.com/1660-4601/18/6/2820/s1: Supplementary Table S1. Factor analysis of food-intake frequency; Supplementary Table S2. Cluster analysis of dietary patterns; Supplementary Table S3. Cognitive function associated with city- and individual-level for older adults in Taiwan 2013–2016 by mixed-effect model (categorical population density).
