# Supplementary material for: Social and Built Environments Related to Cognitive Function of Older Adults: A Multi-Level Analysis Study in Taiwan
Source: Int J Environ Res Public Health. 2021 Mar 10;18(6):2820. doi: 10.3390/ijerph18062820 (PMC7998392; doi:10.3390/ijerph18062820)
Supplement: Supplementary file 1 [file ijerph-18-02820-s001.pdf]

Supplement Table S1. Factor analysis of food-intake frequency

Variables (food items)	Factors (Food Categories)															
	1 White meat and reproduced meat	2 Rice, bread, dairy	3 Melon, bamboo, bean, mushroom	4 Organ blood	5 Cookie, cake, candy	6 Juice, sweet drink	7 Ice cream	8 Light vegetables, root plant	9 Reproduced seafood fermented bean	10 Vegetables	11 Jam, cheese	12 Fruit and pickles	13 Fish eggs, fried	14 Fermented fruit/ vegetables, eggs	15 drinks	16 Milk powder
Fish	**.706**	.023	-.125	.025	-.146	.049	-.058	.135	.115	-.093	-.083	.143	.021	-.020	.064	.077
Poultry	**.663**	.017	.037	.213	.035	.027	.067	.062	.001	-.043	-.035	.077	-.092	-.078	.090	.106
Poultry-lean	**.615**	.057	-.080	.128	.010	.042	.006	.046	-.198	.068	-.094	.034	-.209	.062	.053	.197
Processed see food	**.556**	-.009	.188	.050	.177	-.082	.023	-.010	-.053	.067	.117	-.069	.019	.055	-.177	-.123
Shell fish	**.553**	.091	.316	-.016	.012	.023	-.010	-.089	.152	.030	.058	-.117	.244	-.031	-.040	-.117
Ocean fish	**.547**	.174	.046	-.054	-.121	.046	.022	.223	.130	-.109	-.061	.113	.208	.066	.076	-.010
Other sea food	**.537**	.150	.199	.116	.078	-.007	.039	-.134	.093	.006	.098	-.037	.248	.008	.019	-.196
Processed meat slurry	**.513**	-.147	.169	.078	.182	.006	.175	-.064	-.117	.127	.036	-.118	.004	-.075	-.171	-.030
Processed fish	**.446**	.152	.207	.062	.015	-.073	-.181	.032	.326	.005	.152	-.079	.058	.071	.009	.127
Processed meat	**.356**	-.124	.028	.144	.259	.124	.205	.180	.200	.033	.117	.019	-.047	.124	-.106	-.031
Whole grain rice	-.028	**.656**	.098	-.008	-.032	-.080	-.011	.004	-.082	-.029	-.092	.055	.061	.005	-.028	.004
Whole grain bread	.097	**.642**	.001	.003	.052	.084	-.006	-.067	.102	.046	.084	-.001	-.134	-.030	.028	.058
Yogurt	.079	**.530**	.085	.040	-.089	.239	-.007	-.045	.050	.009	.162	-.198	-.133	-.034	-.096	.113
Nuts	.016	**.517**	.153	.113	.151	-.065	.101	.023	-.032	.083	.085	.193	.148	.021	.048	-.050

Healthy dried fruits	.016	**.471**	.132	.110	.189	-.135	-.015	.159	.089	-.120	.132	-.017	.124	.160	.091	.030
Soy milk	.119	**.354**	.124	.005	.035	.307	.203	-.034	.060	.081	.045	-.081	.033	-.242	.073	-.339
Other soy bean products	.011	.140	**.584**	.064	.084	-.022	-.030	.118	.071	.016	.106	-.065	-.089	-.072	-.019	-.117
Melons	.154	-.030	**.572**	-.022	-.145	.121	.105	.033	-.014	.007	-.011	.105	-.082	.077	.301	.154
Bamboo	.251	.029	**.509**	.089	-.084	-.024	.134	-.093	.093	.057	.052	.123	-.015	.165	.082	.152
Sea plant	.115	.343	**.495**	.052	.061	.038	.004	.149	.045	.049	-.026	.011	.105	-.075	-.039	.067
Pod	.125	.231	**.471**	.002	-.037	.045	.031	.209	.049	.064	-.194	-.058	.025	.191	.157	.031
Fried bean	-.027	.008	**.464**	.252	.269	.077	.068	-.032	-.046	-.091	.092	-.086	-.011	-.174	-.180	-.031
Mushroom	.211	.419	**.430**	-.028	.094	.012	.014	.171	-.027	.112	-.092	.122	.084	.063	-.077	-.054
Liver	.105	.090	.059	**.683**	-.012	.035	-.068	.049	.043	.025	-.046	-.125	-.070	.076	-.015	.044
Other organ	.162	.090	.057	**.649**	.010	.025	.095	-.008	.031	.039	.052	.100	.059	.097	.103	-.142
Other poultry/anima l part	.181	-.011	.012	**.545**	.104	-.004	.123	.034	-.031	-.002	.202	.130	.102	-.072	.015	.055
blood	.170	.038	.192	**.504**	-.002	.070	-.068	-.067	.202	-.028	-.176	-.035	.183	-.081	-.083	.098
Cookies	.048	.073	.154	-.060	**.627**	.017	-.028	-.056	.069	-.008	.019	.213	.098	-.074	.077	.220
Candy	.026	.108	-.047	.110	**.612**	.190	-.055	.143	.116	-.018	-.182	.019	.143	.081	.081	-.075
Cakes	.191	.160	.033	.053	**.539**	.042	.132	.111	-.057	-.006	.177	-.123	-.145	.157	-.017	-.011
Sold juice	-.054	.002	.053	.057	-.021	**.734**	.032	.054	-.029	-.041	-.013	.039	.067	.109	.005	.022
Sweetened drink	.095	.027	.026	.119	.219	**.609**	.122	-.068	.173	.081	-.017	-.020	.065	-.151	.033	-.103
Sweetened milk	.119	.041	.043	-.084	.126	**.436**	-.140	-.049	-.089	-.033	.268	-.058	-.273	.019	-.066	.039
Ice bar	.090	-.029	.104	.053	-.014	-.001	**.687**	-.172	.141	.093	.038	.038	-.083	-.049	.138	-.110
Ice cream	.071	.172	.036	.045	.031	.057	**.649**	.095	-.020	-.140	-.083	-.039	.074	.097	-.162	.142
Pudding and deserts	.075	.086	.212	-.047	.138	.250	**.332**	.000	.297	-.025	.057	-.074	.069	.060	.044	.170

Light vegetables	.152	-.002	.129	.062	.063	-.019	-.058	**.672**	.017	.114	-.012	-.062	-.015	-.142	.005	-.059
Root plants	.160	.174	.299	-.031	.096	.003	-.005	**.580**	.024	.160	-.020	.101	-.064	-.009	-.044	.009
Seafood product	.168	.017	.060	.095	.046	.037	.177	.038	**.669**	.010	.024	.019	-.017	-.070	-.136	.069
Fermented bean	.129	.086	.282	.157	.100	.031	-.043	.016	**.432**	-.039	.158	.016	-.006	.270	-.019	-.102
Vegetables	-.025	-.024	-.011	.042	.000	.084	-.022	.038	.012	**.810**	.061	.002	-.017	.056	.044	.080
Dark vegetables	.085	.115	.185	-.013	-.022	-.112	-.014	.221	-.024	**.639**	-.040	.081	.099	-.016	-.069	-.027
Jam	.056	.176	.058	.027	-.028	.030	-.061	-.083	.133	.063	**.627**	.009	.096	-.028	.033	.004
Cheese	.078	.380	.017	.117	.088	.074	.169	.168	-.033	-.058	**.392**	-.140	-.032	.068	.052	.091
Pure juice	.127	.158	.075	-.075	-.043	-.056	.205	.199	.129	.069	.146	**-.532**	.198	.012	.157	.055
Fruits	.185	.255	.231	-.089	.070	.004	.109	.036	-.042	.104	-.106	**.442**	.096	-.151	-.011	.052
Salted vegetables	.267	.154	-.023	.050	-.014	-.153	.034	.130	.123	.153	.153	**.409**	-.011	.078	.038	.094
Half-fat livestock	.313	-.078	-.048	.179	.067	.084	.112	.177	.172	-.034	.225	**.324**	.114	-.044	.260	-.153
Roe	.175	.064	-.053	.141	.115	.026	-.037	-.078	.009	.082	.061	-.051	**.646**	.028	.046	.082
Fried fast food	.078	.005	.029	.062	-.022	.243	.189	.192	-.246	-.092	.300	.044	**.395**	.139	-.227	.230
Preserves	.120	.160	.090	.003	.288	.024	.132	-.184	.011	.096	-.029	-.049	.102	**.547**	-.011	-.039
Pickles	.091	-.062	.267	.229	.048	.073	-.008	.055	.121	.017	.207	.272	-.060	**.423**	-.136	-.094
Eggs	.282	.115	.146	-.008	.115	.013	.013	.128	.102	.049	.083	.216	-.042	**-.378**	-.018	.138
Other fried food	.127	-.061	.078	.205	.163	.138	.201	.106	-.080	-.116	.182	.148	.194	**-.258**	-.137	-.120
Other drinks	-.014	.031	.097	.032	.091	-.005	-.013	-.027	-.130	-.011	.028	-.060	.022	-.026	**.775**	.015
Milk powder	.096	.147	.085	.010	.083	-.013	.063	-.058	.067	.081	.045	-.003	.094	-.119	.030	**.685**

Note: Extraction Method: Principal Component Analysis. Rotation Method: Quartimax with Kaiser Normalization. Explained variance=49.64%

Supplement Table S2. Cluster analysis of dietary patterns

	Dietary Patterns			
Variables	Cluster 1: Low protein and high vegetables (n=964)	Cluster 2: High protein and high calories (n=244)	Cluster 3: Low vegetable/frits and high cookies/cakes (n=117)	Cluster #: High protein and low vegetables (n=3)
White meat (fish, poultry) and reproduced meat	-.13294	.51124	.01569	.52568
Rice, bread, dairy	-.12355	.49389	-.02468	.49380
Melon, bamboo, bean, mushroom	-.08804	.26633	.16378	.24020
Organ, blood	-.15367	.59324	.05159	-.88420
Cookie, cake, candy	-.07168	.13830	.30234	-.00644
Juice, sweetened drink	-.09661	.38225	.04416	-1.76830
Ice cream	-.00964	.08589	-.11147	.45978
Light vegetable, root plants	.02562	-.01741	-.15448	-.79342
Reproduced seafood, fermented bean	.06238	-.05084	-.40140	-.25660
vegetables	.07337	-.04057	-.08332	-17.02641
Jam, cheese	-.28324	1.10269	.06684	-1.27851
Fruit, sauce vegetable	.05060	-.12623	-.15260	-.04167
Fish egg, fried food	-.13589	.49137	.08589	.35043
Fermented fruit/vegetables, eggs	.02923	-.06641	-.07218	-1.17607
drinks	-.18860	-.40504	2.42245	-.92918
Milk powder	-.06164	.25868	.01171	-1.69001

Note: Analysis by cluster analysis. #The cases in this cluster were too few; the cluster was combined to Cluster 2.

Supplement Table S3. Cognitive function associated with city- and individual-level for older adults in Taiwan 2013-2016 by mixed effect model (categorical population density)

Variable	Model 5a	Model 5b
**Fixed Effects: Individual-level indicators**		
Intercept	7.730 (8.901)	8.344 (5.672)
Age 65-69		1.862 (0.354)***
Age 70-74		1.676 (0.358)***
Age 75-79		1.483 (0.370)***
Sex (Male)		0.370 (0.309)
Marital status (no spouse)		-0.455 (0.262)
Education (ordinal 1-5)		1.296 (0.105)***
Financial satisfaction		0.333 (0.157)*
Smoking (non-smoker)		-0.262 (0.469)
Smoking (Ex-smoker)		0.152 (0.466)
Drinking alcohol (Non-drinker)		0.179 (0.377)
Drinking alcohol (Social drinker)		0.137 (0.362)
Dietary pattern (High protein and high calories)		-0.231 (0.295)
Dietary pattern (low vegetable/fruits and high cookies/drinks)		0.514 (0393)
Physical activity (low)		-0.228 (0.496)
Self-rated health		-0.127 (0.063)*
Disease numbers		0.145 (0.061)*
Physical function		0.200 (0.031)***
Negative emotion		0.002 (0.033)
Positive emotion		0.053 (0.032)
**Fixed Effects: City-level indicators**		
Population density (low)	-0.137 (0.680)	-0.182 (0.419)
Population density (medium)	0.731 (0.634)	0.473 (0.394)
Low income percent	-0.491 (0.210)*	-0.458 (0.130)***
Average household income Gini	-2.236 (11.617)	0.904 (7.296)
Safety in the community	0.176 (0.081)	0.064 (0.051)
Barrier-free sidewalk	2.107 (1.037)	1.117 (0.641)
Elderly abuse rate	4.060 (4.807)	1.984 (3.000)
**Random effect**		
Residual	19.539 (0.851)	13.084 (0.571)
Intercept (city)	0.290 (0.269)	---
**Goodness of fit**	-2 RLL=6242.853	-2RLL=5822.533
	AIC=6246.853	AIC=5826.533
	BIC=6256.798	BIC=5836.443

Note: -2RLL: -2 restricted log likelihood. AIC: Akaike's Information Criterion, BIC: Schwarz's Bayesian Criterion. The reference groups: age (age 80+), sex (female), marital status (having spouse), smoking (current smoker), drinking alcohol (frequent drinker), dietary pattern (low protein and high vegetables), physical activity (medium), and city population density (high). Other variables were continuous or ordinal. #Population density=persons per square kilometres/100, *p<0.05, **p<0.001, ***p<0.001.
